# Consumption of Fallen Umbilical Cord Stump as a Treatment for Infertility: A Case Study from Puducherry

**DOI:** 10.4103/0970-0218.39575

**Published:** 2008-07

**Authors:** T Mahalakshmy, MB Soudarssanane, S Sarkar, S Balaji, B Bharathi, K Talari, J Mahadevan, BK Patel

**Affiliations:** Department of Preventive and Social Medicine, Jawaharlal Institute of Postgraduate Medical Education and Research (JIPMER), Puducherry, India

Infertility is a stigma. The high cost and inaccessibility to treatment turn infertile couples to faith-based alternatives or practices that have been passed down from generation to generation. This report describes a case in which a woman consumed the umbilical cord stump to treat infertility. Initially, this incidence was reported in March 2006 by undergraduate students from the Kurusukuppam area of the Urban Health Centre of the Jawaharlal Institute of Postgraduate Medical Education and Research (JIPMER). This lower-middle class family consisted of RK, his wife G, his mother T and daughters of RK and G. RK was 37 years old, semi-literate and a painter; G was a housewife and educated up to the eighth standard. Six months after their marriage, G became pregnant, but had an abortion in the third month of pregnancy. Thereafter, the couple waited for four years, but G could not conceive. She consulted private allopathic doctors, who prescribed her some medicines without carrying out any investigation/follow-up. Meanwhile, T advised G to consume the umbilical cord stump. In January 2003, G visited Chennai in order to assist her sister-in-law's delivery. Other women present there also advised her to consume the neonate's cord stump as a remedy for her infertility. After a simple ceremony on the eighth day, G consumed a 1-cm-long dried umbilical cord stump embedded in a banana. She conceived the next month. G opined that this sequence of events (pregnancy immediately following the consumption of stump) suggests the curative role of the cord stump. T recalled that 15 years ago, her neighbor had demonstrated the pro-fertility potential of the umbilical cord stump. Both T and her neighbor believed that the stomach (*errai pai*) is connected to the uterus (*garppa pai*), and that the cord had entered the uterus via the stomach, thus inducing pregnancy.

In July 2006, during the community posting for undergraduates, a similar practice was reported in Pullichappallam, Tamil Nadu. A local remedy for infertility in this area was consuming the fallen cord stump. In a focus group discussion (FGD), P (a 40-year-old lady) commented, “If conception does not occur within five months of marriage, we wait for an opportunity to give a fallen cord stump to the ‘infertile’ woman. This practiceis common in our family and has been successful on all occasions.” She had recommended this to three of her relatives, one of whom was infertile for five years. In all instances, conception occurred within a few months. None of them took allopathic treatment. The FGD revealed the belief that the cord ‘donor’ can become infertile; hence, the infertile women should consume it without making the donor aware of it.

Similar practice exists in Kattupalayam and Vannur areas of Tamil Nadu. A newspaper has reported the practice of consuming the products of the third-stage labor for curing infertility in the villages of Thirumangalam taluk of Madurai district, Tamil Nadu.(1) This practice has been documented in the Chenchu tribes of Nallamalai Hills in Andhra Pradesh.(2) They believed that the umbilical cord produces special fertility juice in the infertile woman. Elsewhere, a similar practice has been reported from primitive cultures of Australia, Africa and Hawaii.(3)

This case report brings to light the fact that this practice is not restricted to few families. Reportedly, all known cases have resulted in pregnancy. While we may not share this belief, it highlights the common man's concept about the process of reproduction. Hence, the first step in infertility treatment should be creating awareness about human sexual function and clarify myths associated with infertility since they restrict the afflicted couples from seeking appropriate medical treatment. People's faith in modern medicine should actually improve when infertility treatments become more accessible and affordable. However, if a series of cases such as the abovementioned incidents occur, it may then become necessary to epidemiologically study such interventions.
